# Supplementary material for: Single influenza A viruses induce nanoscale cellular reprogramming at the virus-cell interface
Source: Nat Commun. 2025 Apr 25;16:3846. doi: 10.1038/s41467-025-58935-8 (PMC12032206; doi:10.1038/s41467-025-58935-8)
Supplement: Supplementary file 1 — Supplementary Information [file 41467_2025_58935_MOESM1_ESM.pdf]

Supplementary Material

Single influenza A viruses induce nanoscale cellular reprogramming at the virus-cell interface

Lukas Broich[1], Hannah Wullenkord[2], Maria Kaukab Osman[3,4,5], Yang Fu[1], Mathias Müsken[6], Peter Reuther[3,4], Mark Brönstrup[2,7,8], Christian Sieben[1,9,*]

[1] Nanoscale Infection Biology Group, Helmholtz Centre for Infection Research, Braunschweig, Germany

[2] Department of Chemical Biology, Helmholtz Centre for Infection Research, Braunschweig, Germany

[3] Institute of Virology, Medical Center - University of Freiburg, Freiburg, Germany

[4] Faculty of Medicine, University of Freiburg, Freiburg, Germany

[5] Spemann Graduate School of Biology and Medicine, University of Freiburg, Freiburg, Germany

[6] Central Facility for Microscopy, Helmholtz Centre for Infection Research, Braunschweig, Germany

[7] German Center for Infection Research (DZIF), Site Hannover-Braunschweig, Braunschweig, Germany

[8] Institute of Organic Chemistry, Leibniz University Hannover, Hannover, Germany

[9] Institute of Genetics, Technische Universität Braunschweig, Braunschweig, Germany

[*] correspondence:

Christian Sieben
Helmholtz Centre for Infection Research
Nanoscale Infection Biology
Inhoffenstr. 7
38124 Braunschweig
Germany
Tel: +49 531 6181 2950
E-Mail: christian.sieben@helmholtz-hzi.de

Keywords: influenza, virus entry, endocytosis, single-molecule tracking, super-resolution microscopy

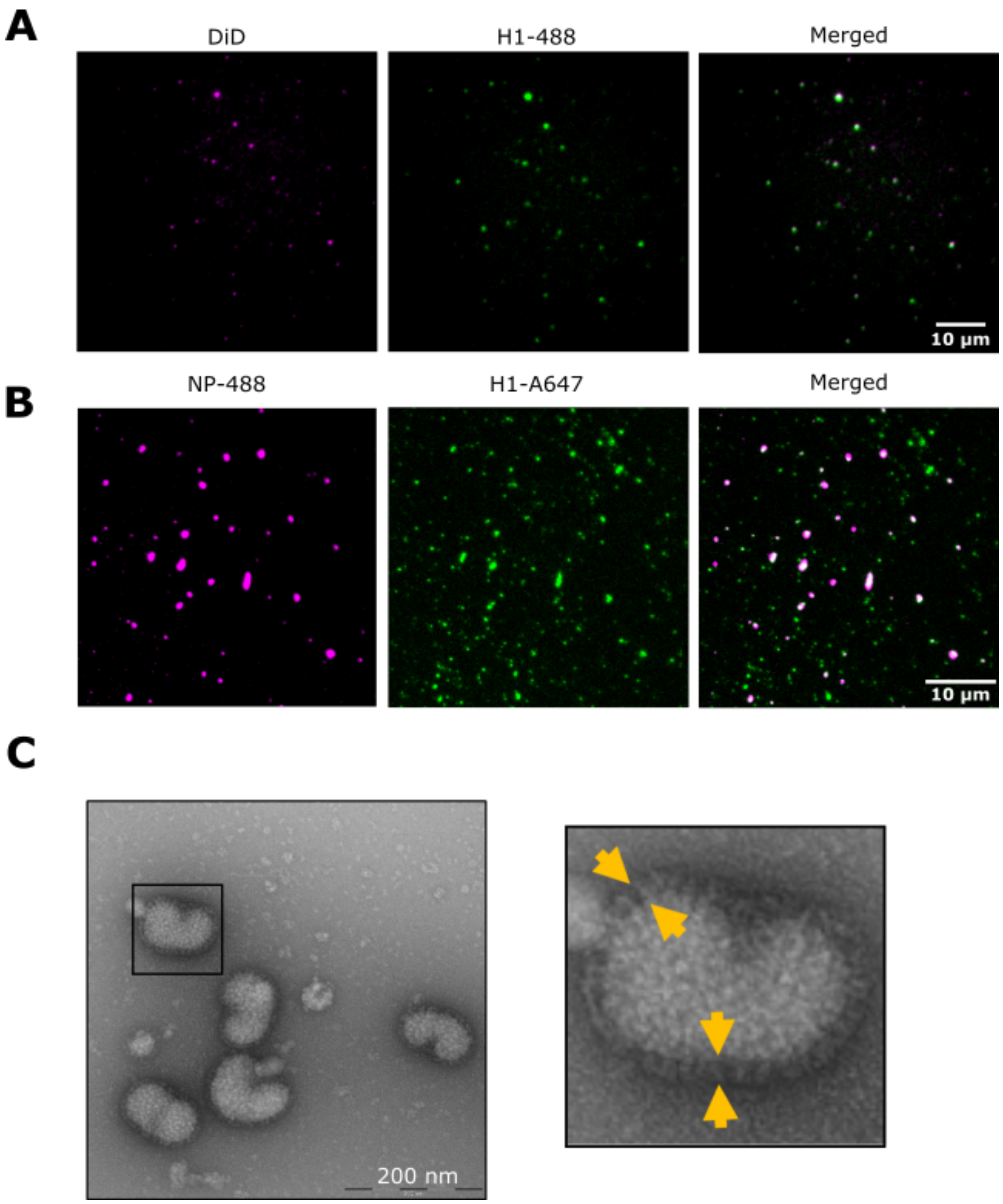

Figure S1 | Quality control of DID-labelled IAV particles. To ensure that DID-labelled particles used in all single-virus experiments represent intact IAVs, we immobilized IAVs after DID labeling and size exclusion purification on microscopy glass slides. We then immunostained the viral particles using anti-H1 only (A) or anti-NP and anti-H1 antibodies (B). While we found that the majority of DID-labelled particles colocalize with the anti-H1N1 signal, the overlap between anti-H1 and anti-NP was lower, indicating free IAV nucleoprotein in our virus preparation. We further confirmed intact spike-decorated (yellow arrows) virions in our preparation using negative stain transmission electron microscopy (C).

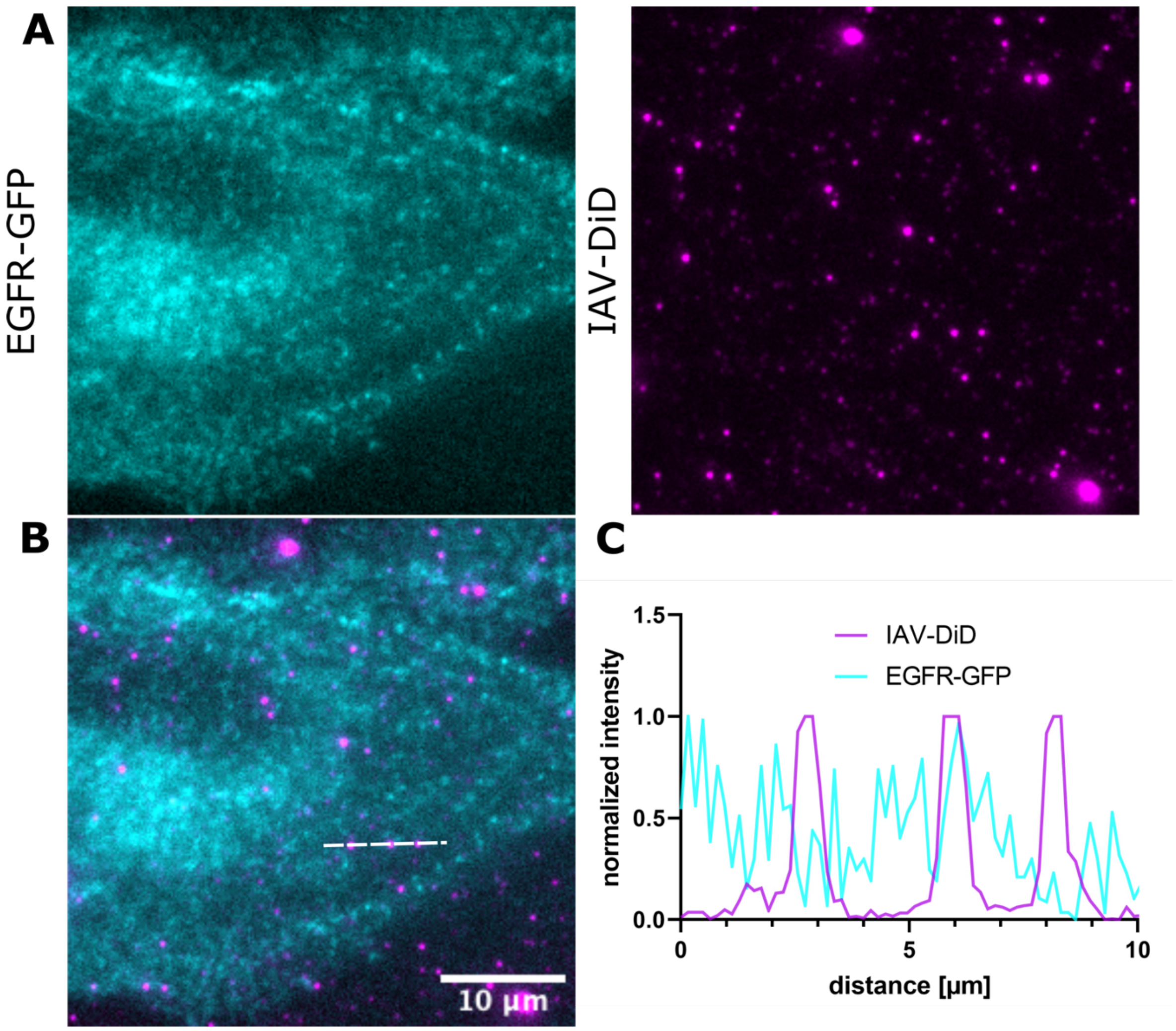

Figure S2 | EGFR-GFP did not colocalize with IAV-DID after sialidase treatment. A549 cells stably expressing EGFR-GFP (A) were cultivated in top of immobilized DID-labelled IAV PR8 (B). Before imaging, the cells were treated with 250 mU/ml sialidase for 1h. A line plot measurement (white line in B) shows no accumulation of EGFR-GFP at the virus-binding site (C). Images are representative. The experiment was repeated twice with similar results. Source data are provided as a Source Data file.

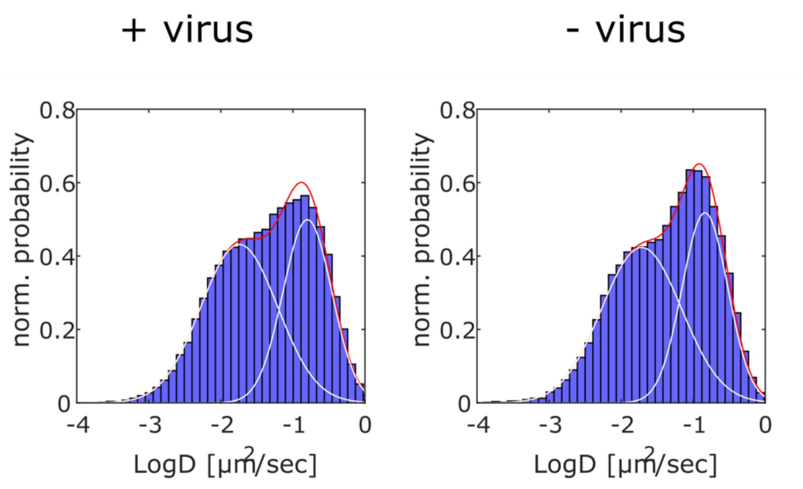

Figure S3 | Histogram of EGFR diffusivity in cells cultivated with or without immobilized IAV. A549 cells stably expressing EGFR-mEos3.2 were cultivated with or without immobilized IAV PR8. Histograms of the measured diffusion coefficients are shown. The scaled probability densities could be well fitted by two Gaussians (red curves). The white curves show the two subpopulations: left (right) for relatively immobile (mobile) tracks. Source data are provided as a Source Data file.

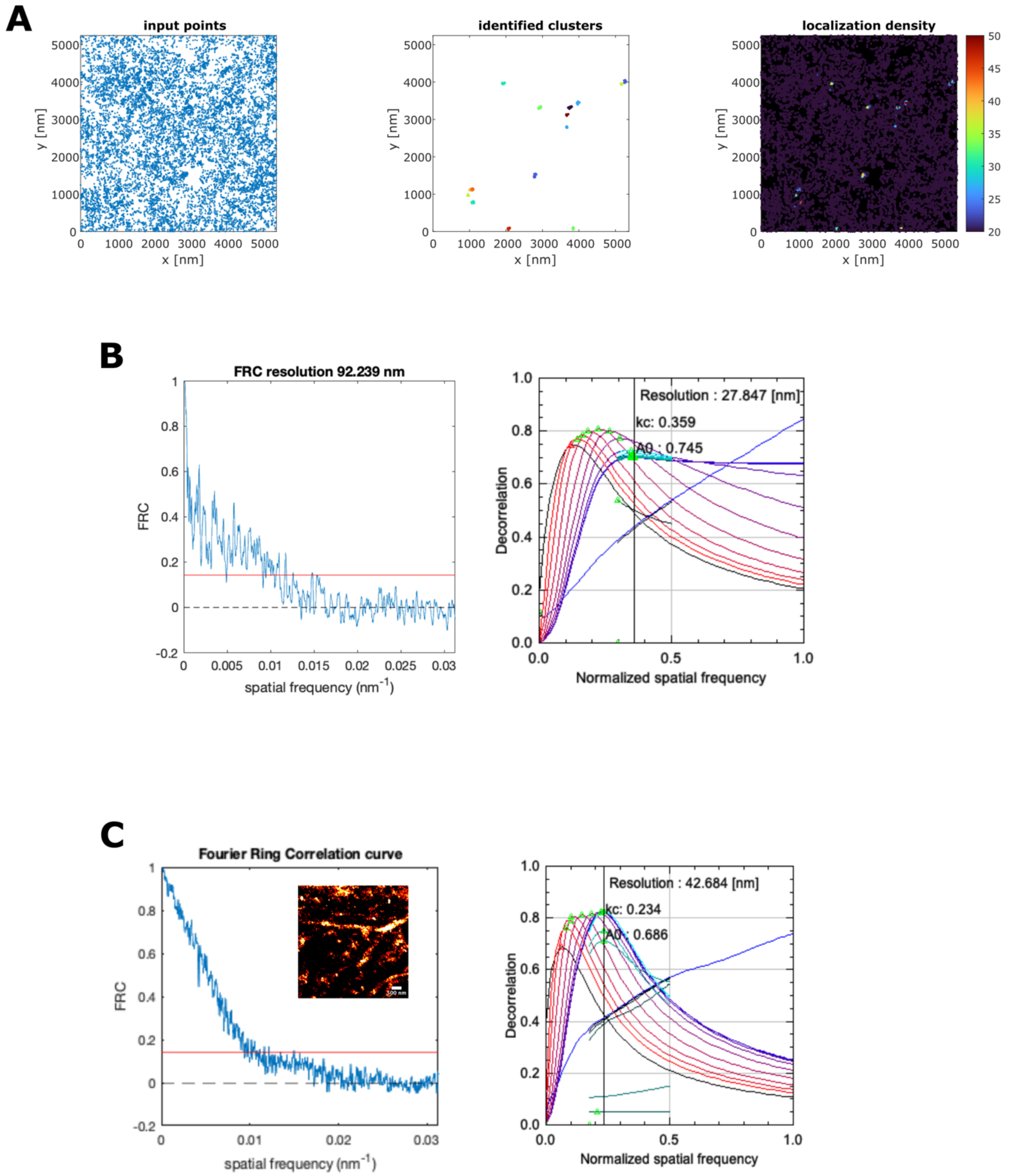

Figure S4 | Cluster analysis and resolution estimation of single-molecule localization data. Live A549 cells stably expressing EGFR-mEos3.2 were imaged using TIRF illumination (A, as also presented in Fig. 2B). Exemplary localization data and extracted clusters are shown in (A), together with a plot showing the local localization density color-coded (A, right side). The localization density was calculated for each localization as the number of other localizations within a radius of 30 nm (1.5-fold lateral localization precision) to aid the visual identification of clusters. Cluster analysis is described in the methods section, the results are summarized in Figure S5. We further analyzed the resolution of our localization datasets using Fourier ring correlation[1] and image decorrelation[2]. (B) shows the resolution analysis of the localization data shown in (A). (C) shows the resolution analysis of the STORM localization data shown in Fig. 4B. FRC analysis of the area shown in (C, inset) revealed 91 nm.

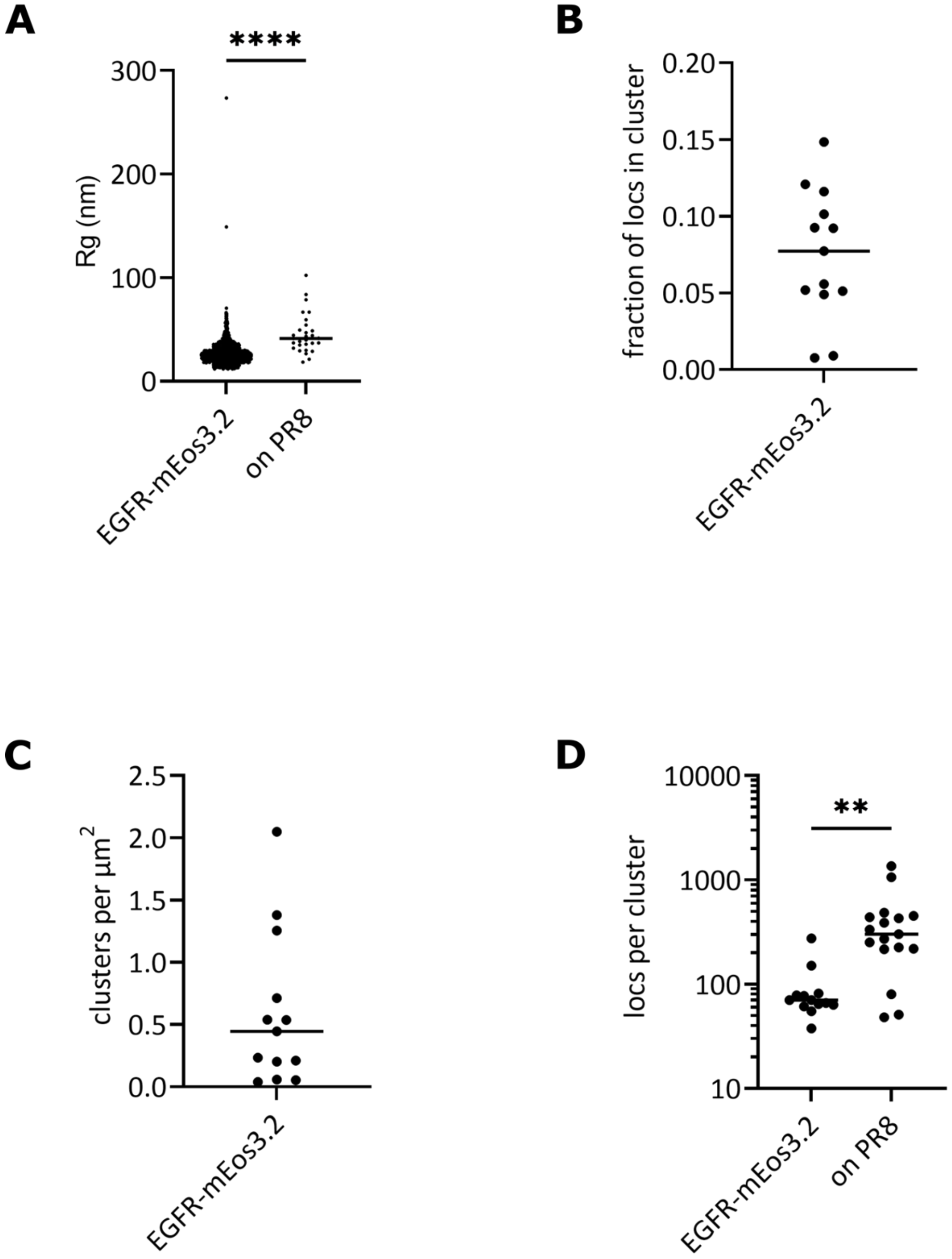

Figure S5 | Summary of DBSCAN-based cluster analysis of EGFR-mEos3.2 imaged in live A549 cells. Live A549 cells stably expressing EGFR-mEos3.2 were imaged using TIRF illumination (as also presented in Fig. 2B). Localization and cluster analysis were performed as described in the methods section. (**A**, each point represents one cluster, vertical line shows the mean, unpaired t test, $p<0.0001$). The mean radius of gyration (Rg) of EGFR clusters was 25.2 nm (top left plot), similar to our earlier findings[3]. Clusters found on immobilized IAV/PR8 particles were larger with a mean Rg of 45.1 nm. Further cluster statistics are shown in **C-D**. **C-B**, each point represents one cell. The vertical line shows the mean. **D**, each point represents one cluster, vertical line shows the mean. Without virus, we detected on average 88 localizations per cluster (**D,** unpaired t test, $p<0.01$). Clusters found on immobilized IAV/PR8 particles contained on average 388 localizations. n = 6 biological replicates from two experiments. Source data are provided as a Source Data file.

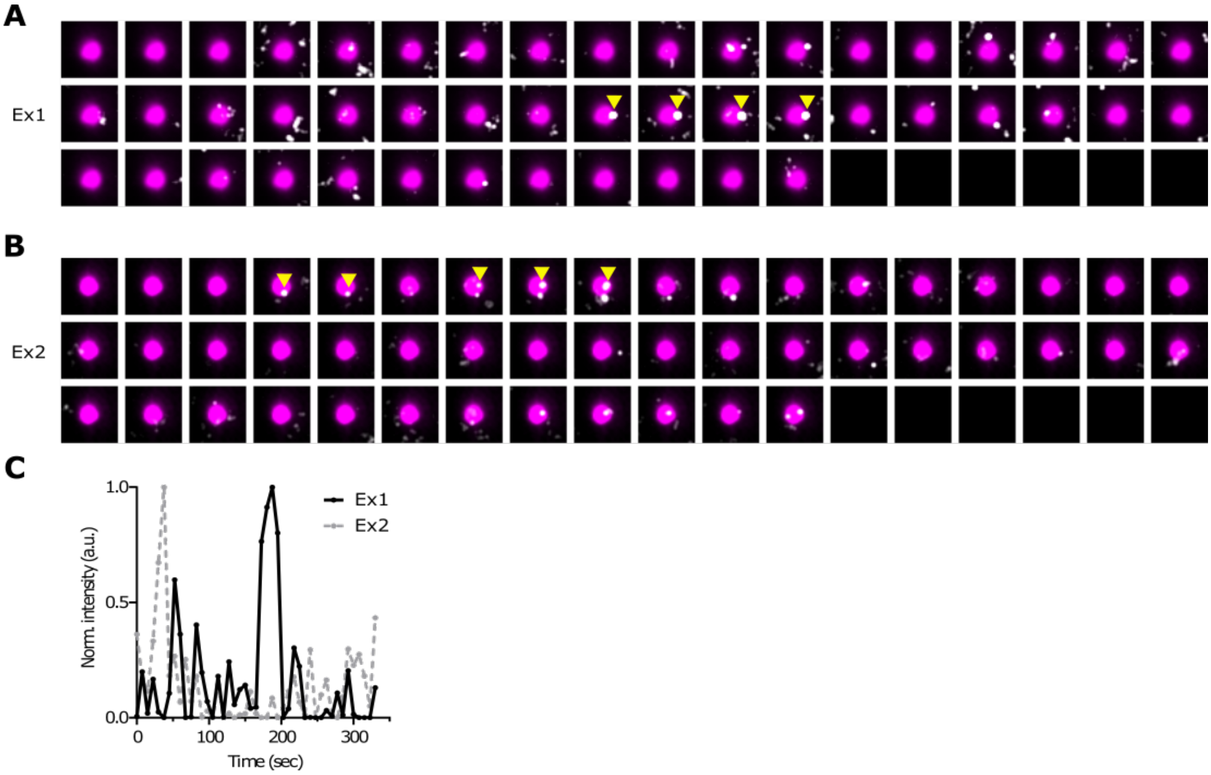

Figure S6 | Time-binned rendering of EGFR-mEos3.2 localizations from a live-cell acquisition. EGFR-mEos3.2 localizations from a live-cell acquisition were rendered using 6 sec time binning (200 frames) resulting in 50 reconstructions across a 5 min acquisition. The position of the respective labelled IAV particle is shown in magenta, the rendered PALM reconstructions in grey. Recurrent appearance of EGFR clusters (yellow arrow heads) can be observed indicating dynamic exchange of EGFR between the virus-cell interface and the remaining plasma membrane. (A) and (B) show two examples. (C) shows the development of the integrated intensity of the rendered EGFR PALM localizations over time. Source data are provided as a Source Data file.

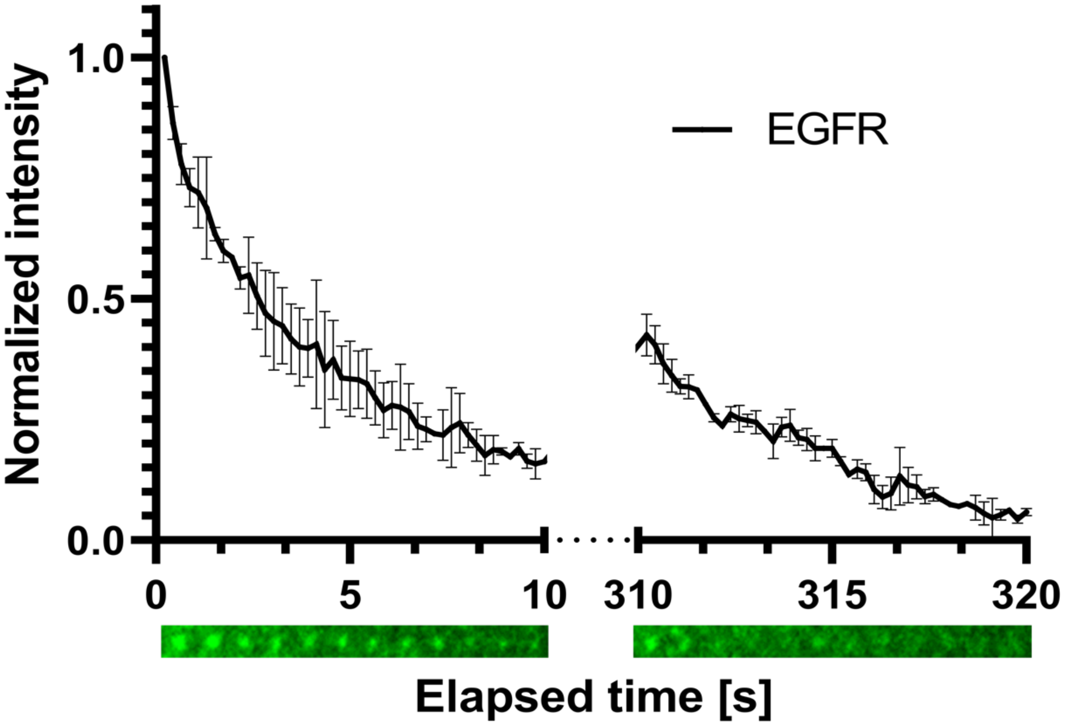

Figure S7 | EGFR-GFP accumulated at the virus-binding site bleaches and recovers over time. A549 cells stably expressing EGFR-GFP were cultivated in top of DiD-labelled IAV PR8. Interaction between IAV and EGFR led to an accumulation of EGFR-GFP at the virus-binding site (shown in **Figure 2A**). Continuous imaging of the same area led to photobleaching of the GFP signal (left side), which recovered after the excitation laser was switched of for 300 sec (right side). n = 3 biological replicates from two experiments. Data are presented as mean values ± SD. Source data are provided as a Source Data file.

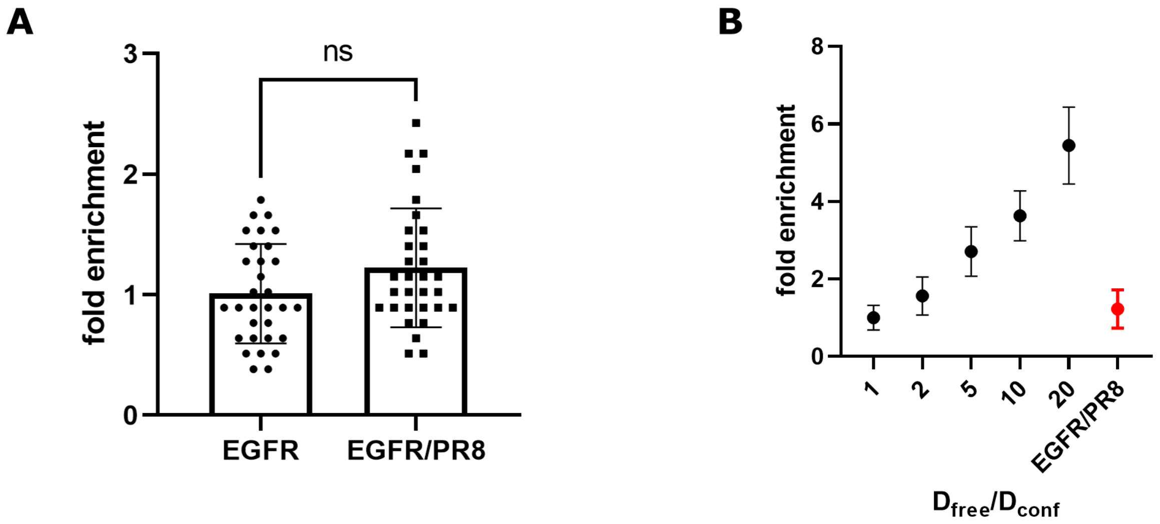

Figure S8 | Virus-induced diffusion slowdown leads to local enrichment of receptor proteins. To calculate if a slowdown of receptor proteins at the virus binding site leads to local protein enrichment, we simulated the 2D random diffusion of EGFR using a diffusion coefficient of D_{EGFR} = 0.050 µm^2/s. We then added circular regions (r = 50 nm) to the simulation, where the proteins were slowed down according to our PALM measurements to $D_{EGFR\text{-}PR8}$ = 0.024 µm^2/s. We simulated 4000 time steps with 30 ms interval. At the end, we examined the number of receptors associated with the circular regions (i.e., viruses). (**A**) For the IAV PR8-mediated diffusion slowdown measured for EGFR, we find a moderate non-significant local enrichment as compared to freely diffusing receptors. (**B**) We also simulated stronger local slowdown as possible for other IAV-receptor combinations, which indeed leads to more pronounced local protein enrichment. (**A, B**) n = 30 simulation runs. Data are presented as mean values ± SD. Unpaired t test, p>0.1. Source data are provided as a Source Data file.

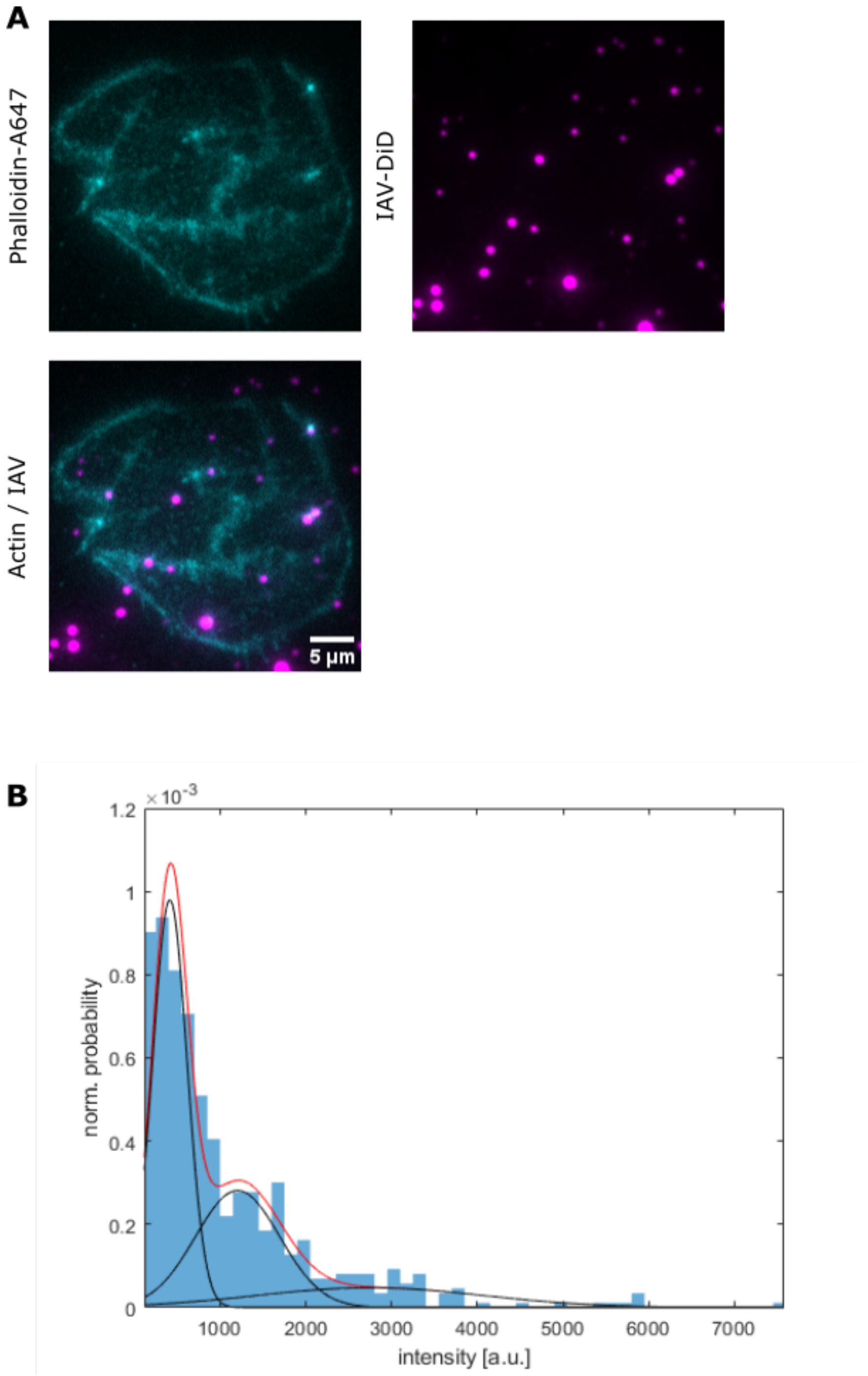

Figure S9 | Immobilized IAV colocalize with phalloidin-Alexa647 in A549 cells. A549 cells were cultivated on top of immobilized DiD-labelled IAV PR8. The cells were fixed and labelled using phalloidin conjugated with Alexa647 and imaged using TIRF illumination (**A**). For quantification, the viruses were detected automatically and the phalloidin-Alexa647 signal analyzed at the respective locations. The intensity distribution can be fitted using the sum of three Gaussians (B, red line). The three populations (B, black curves) were ascribed as 1) not below cells, 2) phalloidin low and 3) phalloidin high. Source data are provided as a Source Data file.

References

1 Nieuwenhuizen, R. P. *et al.* Measuring image resolution in optical nanoscopy. *Nat Methods* **10**, 557-562 (2013). https://doi.org:10.1038/nmeth.2448

2 Descloux, A., Grussmayer, K. S. & Radenovic, A. Parameter-free image resolution estimation based on decorrelation analysis. *Nat Methods* **16**, 918-924 (2019). https://doi.org:10.1038/s41592-019-0515-7

3 Sieben, C., Sezgin, E., Eggeling, C. & Manley, S. Influenza A viruses use multivalent sialic acid clusters for cell binding and receptor activation. *PLoS Pathog* **16**, e1008656 (2020). https://doi.org:10.1371/journal.ppat.1008656
