# Supplementary material for: Single influenza A viruses induce nanoscale cellular reprogramming at the virus-cell interface
Source: Nat Commun. 2025 Apr 25;16:3846. doi: 10.1038/s41467-025-58935-8 (PMC12032206; doi:10.1038/s41467-025-58935-8)
Supplement: Supplementary file 2 — Description of Additional Supplementary Files [file 41467_2025_58935_MOESM2_ESM.docx]

**File Name:** Supplementary Video 1

**Description:** Time-binned PALM super-resolution movie of EGFR dynamics at the virus-binding site. EGFR-mEos3.2 localizations from a live-cell acquisition were rendered using 6 sec time binning. The position of the labelled IAV particle is shown in magenta. Recurrent appearance of EGFR clusters can be observed indicating dynamic exchanges of EGFR between the virus-interface and the remaining plasma membrane.

**File Name:** Supplementary Video 2

**Description:** Time-binned PALM super-resolution movie of EGFR dynamics at the virus-binding site. EGFR-mEos3.2 localizations from a live-cell acquisition were rendered using 6 sec time binning. The position of the labelled IAV particle is shown in magenta. The recurrent appearance of EGFR clusters can be observed indicating dynamic exchanges of EGFR between the virus-interface and the remaining plasma membrane.

**File Name:** Supplementary Video 3

**Description:** TIRF live-cell imaging of A549 cells expressing LifeAct-GFP cultivated on top of immobilized IAV. A549 cells expressing LifeAct-GFP (cyan) were cultivated in top of immobilized DiD-labelled IAV PR8 and imaged by TIRF.
