# Supplementary figures and images for: Single influenza A viruses induce nanoscale cellular reprogramming at the virus-cell interface
Source: Nat Commun. 2025 Apr 25;16:3846. doi: 10.1038/s41467-025-58935-8 (PMC12032206; doi:10.1038/s41467-025-58935-8)

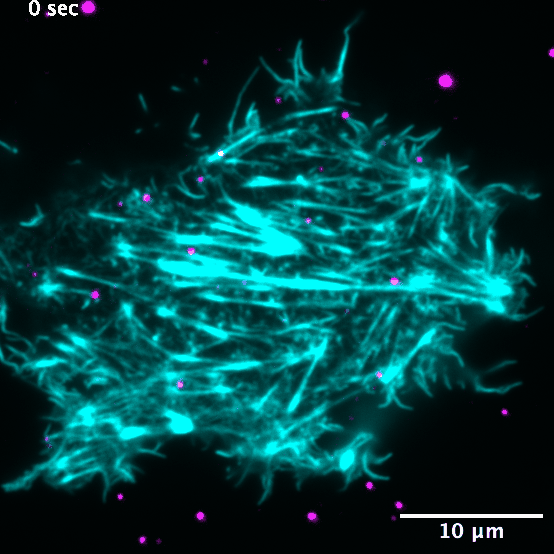

Supplement: Supplementary file 5 — Supplementary Movie 3 [file 41467_2025_58935_MOESM5_ESM.gif]
