# Supplementary material for: Single influenza A viruses induce nanoscale cellular reprogramming at the virus-cell interface
Source: Nat Commun. 2025 Apr 25;16:3846. doi: 10.1038/s41467-025-58935-8 (PMC12032206; doi:10.1038/s41467-025-58935-8)
Supplement: Supplementary file 6 — Reporting Summary [file 41467_2025_58935_MOESM6_ESM.pdf]

nature portfolio

Corresponding author(s): Christian Sieben

Last updated by author(s): Mar 12, 2025

Reporting Summary

Nature Portfolio wishes to improve the reproducibility of the work that we publish. This form provides structure for consistency and transparency in reporting. For further information on Nature Portfolio policies, see our Editorial Policies and the Editorial Policy Checklist.

Statistics

For all statistical analyses, confirm that the following items are present in the figure legend, table legend, main text, or Methods section.

n/a	Confirmed	
☐	☒	The exact sample size (*n*) for each experimental group/condition, given as a discrete number and unit of measurement
☐	☒	A statement on whether measurements were taken from distinct samples or whether the same sample was measured repeatedly
☐	☒	The statistical test(s) used AND whether they are one- or two-sided<br>*Only common tests should be described solely by name; describe more complex techniques in the Methods section.*
☒	☐	A description of all covariates tested
☐	☒	A description of any assumptions or corrections, such as tests of normality and adjustment for multiple comparisons
☐	☒	A full description of the statistical parameters including central tendency (e.g. means) or other basic estimates (e.g. regression coefficient) AND variation (e.g. standard deviation) or associated estimates of uncertainty (e.g. confidence intervals)
☐	☒	For null hypothesis testing, the test statistic (e.g. *F*, *t*, *r*) with confidence intervals, effect sizes, degrees of freedom and *P* value noted<br>*Give P values as exact values whenever suitable.*
☒	☐	For Bayesian analysis, information on the choice of priors and Markov chain Monte Carlo settings
☒	☐	For hierarchical and complex designs, identification of the appropriate level for tests and full reporting of outcomes
☒	☐	Estimates of effect sizes (e.g. Cohen's *d*, Pearson's *r*), indicating how they were calculated

Our web collection on statistics for biologists contains articles on many of the points above.

Software and code

Policy information about availability of computer code

Data collection	NIS Elements AR 5.11.01 (Nikon), Keyence contoller software
Data analysis	Decode v0.1, Matlab 2020b, Fiji with ImageJ 1.53q, ThunderSTORM v1.3, previously used custom processing codes (10.5281/zenodo.15005579), Python, TrackPy, CellProfiler,

For manuscripts utilizing custom algorithms or software that are central to the research but not yet described in published literature, software must be made available to editors and reviewers. We strongly encourage code deposition in a community repository (e.g. GitHub). See the Nature Portfolio guidelines for submitting code & software for further information.

Data

Policy information about availability of data

All manuscripts must include a data availability statement. This statement should provide the following information, where applicable:

- Accession codes, unique identifiers, or web links for publicly available datasets
- A description of any restrictions on data availability
- For clinical datasets or third party data, please ensure that the statement adheres to our policy

Source data are provided with this paper. Example datasets are available via https://zenodo.org/records/14222289.

Research involving human participants, their data, or biological material

Policy information about studies with human participants or human data. See also policy information about sex, gender (identity/presentation), and sexual orientation and race, ethnicity and racism.

Reporting on sex and gender	n/a
Reporting on race, ethnicity, or other socially relevant groupings	n/a
Population characteristics	n/a
Recruitment	n/a
Ethics oversight	n/a

Note that full information on the approval of the study protocol must also be provided in the manuscript.

Field-specific reporting

Please select the one below that is the best fit for your research. If you are not sure, read the appropriate sections before making your selection.

[x] Life sciences [ ] Behavioural & social sciences [ ] Ecological, evolutionary & environmental sciences

For a reference copy of the document with all sections, see nature.com/documents/nr-reporting-summary-flat.pdf

Life sciences study design

All studies must disclose on these points even when the disclosure is negative.

Sample size	No statistical method was used to determine sample sizes and no sample size calculation was performed. Sample sizes were chosen to match our previous published work in which similar experimental setups were used (Sieben, Plos Path, 2020; Glueck, iScience, 2023; Degen, Nature, 2023).
Data exclusions	No data were excluded from the analysis.
Replication	Each experiment was repeated as described in the figure legends. Each replicable experiment was repeated at least three times independently. The number of replicate performed for each figure is stated in the figure legends.
Randomization	There was no randomization for these experiments. This study is not a randomised control trial and randomisation is not conventionally used in in vitro/in cellulo studies such as this one. All groups of experiments were performed using the same experimental conditions and protocols.
Blinding	Assays used objective quantification methods that are not susceptible to bias, so samples were not blinded.

Reporting for specific materials, systems and methods

We require information from authors about some types of materials, experimental systems and methods used in many studies. Here, indicate whether each material, system or method listed is relevant to your study. If you are not sure if a list item applies to your research, read the appropriate section before selecting a response.

Materials & experimental systems

n/a	Involved in the study	
[ ]	[x]	Antibodies
[ ]	[x]	Eukaryotic cell lines
[x]	[ ]	Palaeontology and archaeology
[x]	[ ]	Animals and other organisms
[x]	[ ]	Clinical data
[x]	[ ]	Dual use research of concern
[x]	[ ]	Plants

Methods

n/a	Involved in the study	
[x]	[ ]	ChIP-seq
[x]	[ ]	Flow cytometry
[x]	[ ]	MRI-based neuroimaging

Antibodies

Antibodies used	Anti-NP (Merck, MAB8257), anti-H1 (Thermo Fischer, PA534929)

Validation	Both antibodies have been validated by their manufacturer and are extensively used in the scientific community.

Eukaryotic cell lines

Policy information about cell lines and Sex and Gender in Research

Cell line source(s)	A549 cells were obtained from DSMZ (ACC 107); MDA-MB-231 cells were obtained from Drubins Laboratory, UC Berkeley, USA
Authentication	The cell lines used in this study have not been authenticated by the authors.
Mycoplasma contamination	The used cell lines are routinely tested for mycoplasma contamination and cells are typically discarded if positive.
Commonly misidentified lines (See ICLAC register)	We did not use commonly misidentified cell lines.

Plants

Seed stocks	n/a
Novel plant genotypes	n/a
Authentication	n/a
