# Supplementary material for: Single influenza A viruses induce nanoscale cellular reprogramming at the virus-cell interface
Source: Nat Commun. 2025 Apr 25;16:3846. doi: 10.1038/s41467-025-58935-8 (PMC12032206; doi:10.1038/s41467-025-58935-8)
Supplement: Supplementary file 7 — Transparent Peer Review file [file 41467_2025_58935_MOESM7_ESM.pdf]

nature portfolio

Peer Review File

Single influenza A viruses induce nanoscale cellular reprogramming at the virus-cell interface

Corresponding Author: Professor Christian Sieben

Editorial Note: Parts of this peer review file have been redacted as indicated to maintain the confidentiality unpublished data.

This file contains all reviewer reports in order by version, followed by all author rebuttals in order by version.

Version 0:

Reviewer comments:

Reviewer #1

(Remarks to the Author)
In this paper Broich and co-workers suggest that IAV induces nanoscale cellular reprogramming when engaging/priming with cellular receptors, having an impact on AP-2 and actin dynamics. Both issues seem to be reasonable provided that IAV needs to get inside the cell via clathrin mediated endocytosis to reach late endosomes and acidic pH.

The authors implement a method to immobilize IAV viruses employing a glass substrate modified with silane PEG NHS na d silane PEG (Figure 1) assuming that this way, one could recover the interactions between IAV receptros and AF (or other factors) and characterize them since, they are transient. The authors immobilize IAV viruses labelled with DiD. The same criticism made on the accompanying paper regarding the true nature of IAV and DiD not being the best marker for viruses (as it stains every vesicle, exosome or junk tht contains lipids) can be made here. I assume there is a subset of IAV particles that are also labelled with DiD but one cannot be sure unless staining both, the receptors and a viral component (matrix M1?). The different sizes for these particles could account on the fact that one can image aggregates, exosomes, viral IAV particles and junk that contains IAV receptors.

In Figure 2, one can measure with super-resolution how EGFR accumulates around these particles, when tracking those employing MSD a bimodal distribution (mobile and immobile is seen). Not sure how these, or other receptors will adapt to particles decorated with different IAV receptors. In here, it is possible that the degree of incorporation plays a role. One needs to perform these experiments with IAV particles that are productive and one can recognize before hand. Technically, these experiments are of high complexity and seem to be performed really well and with care.

The TIRF data shown in Figure 3 is also of high calibre. However, what these data show is that most likely, any particle decorated with IAV receptors will induce AP2 recruitment and will initiate CME; which in turn will have an impact on actin rearrangement (Figure 4). Also, even if the authors can show this for real IAV particles able to fuse in late endosomes, these facts were previously described with SVT.

Overall, the experiments in this paper are very well performed and the microscope approaches, together with image analysis and stats are very nice and rigorous. However, I think one needs to be certain that one is imaging real IAV particles. Also, it would be of interest to follow these particles with WF and check how they are entering the host, and if some of them are fusogenic at all. By combining DiD labelling with internal markers one should be able to tell which of these particles is undergoing hemifusion and full fusion as has been shown by others in the past.

Reviewer #2

(Remarks to the Author)
The papers Christian Sieben describe the nanoscale clusters of influenza A virus receptors upon HA binding.

The data is compelling and of interest to a broad readership. The microscopy approaches are fantastic. Although the first manuscript is of a technical complexity, probably beyond a part of that broad readership. The capture of live cells using both virus and fibronectin comes across more as a trick, without real biological consequences. Would there be fluorescently labelled glycosylated membrane proteins that are not clustered upon engagement of the virus?

This methodology was then applied to H18N11 in an accompanying paper. This virus uses a protein as a receptor and thus rules of engagement could be different, but in the end was not. In this paper the interaction of H18 and different MHC construct where first studied in detail before state-of-the-art microscopy approaches were used to dissect receptor clustering. Due to the availability of a null-mutant receptor protein clustering was convincing.

Eventually clustering of receptors appears to be quite universal, and this could be discussed more. It also decreases the novelty somewhat as it is quite expected and the technological advancements is then the novelty here.

Single IAVs induces Nanoscale cellular reprogramming at the virus-cell interface
Major
-The introduction steers towards a protein receptor, yet these are proteins that migrate from and to the plasma membrane, sialylated glycans on those proteins are the receptors. This change in premise should be be accounted for.
-Fig 1D, sure viruses are located under the cell, but also next to it, there is no data for actual attachment as this could just be due the the fibronectin peptide (which by it self is a smart move). The concluding text of this paragraph should be toned down.
-The same holds true for the conclusion of the next “chapter”, it is not known if this cluster is “functional”. Again, probably glycans on EGFR are bound so it should say capable of clustering EGFR or something along that line.
-Only 15% of the viruses colocalize with actin? Is this a lot? What does this mean? It is gleaned over it, I also don’t get the arc, blobs and holes classification.

Minor
-IAV viral envelope proteins are not spikes…
-sialylated glycans are receptors and not attachment factors, please change throughout
-the graphs Fig 1 B and C should be discussed and referenced in the text better, I had to go back and forth many times.
-Why would you compared fluorescent beads with fluorescent beads on glass surfaces?! Just that the coating is efficient?
-Because AP2 is reqruited it can also still be dispensable, so this is not so surprising.
-First line of the discussion   reqruitment of glycoproteins

The bat IAV H18N11 induces nanoscale MHCII clustering upon host cell attachment
Major
- “inverse infection” while there is no infection, just binding…
-Was the addition of the fibronectin peptie necessary here as well?! This should be described in an accompanying paper.

Minor
-discussion, bat and avian IAV both cluster a receptor for endocytosis is conserved for other viruses as well… this could be further discussed
-hardly any information is given in the methods for the creation of plasmids for the soluble expression of MHC proteins
-same goes for the inverse binding assay how where these viruses coated the glas slides and how was this controlled?

Reviewer #3

(Remarks to the Author)
Review Report:

The paper titled, “ Single Influenza A virus induce nanoscale …. ” by Broich et al studies virus-receptor interaction and reports that viral receptors are not immobilized by the virus. They report that this process further leads to the accumulation of local receptor. The authors claim to study these processes using TIRF-STORM super-resolution microscopy. To my understanding, this is the only novel feature of the present work. Here are my major comments:

1. The statement, “ … preserving the viruses’ native state for uncompromised cell interaction ” is not substantiated.
2. The statement, “ … viral receptors are not immobilized by the virus but rather slowed down … ” is not supported by imaging data.
3. Note that, the dynamic response requires time-lapse super-resolutiom imaging and related single molecule analysis.
4. Studies carried out in the literature related to super-resolution study of Influenza A virus is completely ignored. Please see the following papers that clearly indicate clustering study of HA in cellular system.
- Nikki M. Curthoys et al., Influenza Hemagglutinin Modulates Phosphatidylinositol 4,5-Bisphosphate Membrane Clustering, Biophys J . 116, 893-909 (2019).
- S T Hess et al., Dynamic clustered distribution of hemagglutinin resolved at 40 nm in living cell membranes discriminates between raft theories, PNAS 104, 17370-17375 (2007).
- Raut P, Obeng B, Waters H, Zimmerberg J, Gosse JA, Hess ST., Phosphatidylinositol 4,5-Bisphosphate Mediates the Co-Distribution of Influenza A Hemagglutinin and Matrix Protein M1 at the Plasma Membrane. Viruses. 14, 2509 (2022).

5. On page #108, it is state that, “… indicating a direct and quantifiable interaction between receptor and virus”. This is not demonstrated well.

6. In the super-resolution study, a lot of information is missing such as, localization plot, Fourier Ring Correlation, drift-correction etc.
7. The characteristics of local reporter accumulation such as, size of accumulates, its density, number per cluster and fraction of accumulated reporters need to be determined.

Based on the above and my reading of the manuscript suggests that the manuscript is not mature enough for publication. More work along with single molecule analysis need to be done for drawing the conclusions. In addition, the authors need to look at reported super-resolution study on Influenza A virus.

Version 1:

Reviewer comments:

Reviewer #1

(Remarks to the Author)
The authors have performed immunostaining and transmission electron microscopy of the fluorescently-labelled IAVs to evaluate the quality of our virus preparation.
It seems that a lot of the DID-labelled particles stained positive for H1 and some of them contained nucleoprotein (NP), confirming the presence of intact and H1 decorated virions.

This was my main criticism and the authors have taken care of it, therefore I support the publication of this article in its current form.

(Remarks on code availability)

Reviewer #2

(Remarks to the Author)
The authors sufficiently replied to my suggestions / comments

(Remarks on code availability)

Reviewer #3

(Remarks to the Author)
My comments:

In response 2, what needs to be done is to carry out additional experiments to support the statement, which is clearly not done. Just changing the wording is not good enough. Even the new statement, "viral receptor show reduced mobility at the virus binding site " needs to be substantiated with experimental imaging data.
Response 3 requires time-lapse super-resolution data, which is not provided. This is essential for understanding dynamic response (how single molecules comes together to form clusters and the build-up of cluster) during the observation time. Unfortunately this is not provided.
In response 5, the systematic study using different IAV strains how HA-receptor affinity modulates the three cluster metrics should have been carried out. This is essential for the present study and the claims in this study.

In response 6, the clusters are hardly visible from non-clustered molecules (see, Fig. S4A). In general, the clusters come more prominently and are quite distinct (see, the clusters in Ref. Nikki M. Curthoys et al., Biophys J . 116, 893-909 (2019).). What is needed is probably large incubation time for large clusters. The study needs further investigation.

(Remarks on code availability)

Version 2:

Reviewer comments:

Reviewer #1

(Remarks to the Author)
I have carefully read the comments for other reviewer's reticences and consider that the response from the authors is accurate and describes well the results.

They also describe the limitations of sptPALM and explain how difficult it is to measure simultaneously molecular cluster dynamics in difracted limited spots (such as single viruses).

I am happy with the authors' response.

(Remarks on code availability)

Open Access This Peer Review File is licensed under a Creative Commons Attribution 4.0 International License, which permits use, sharing, adaptation, distribution and reproduction in any medium or format, as long as you give appropriate credit to the original author(s) and the source, provide a link to the Creative Commons license, and indicate if changes were made.
In cases where reviewers are anonymous, credit should be given to 'Anonymous Referee' and the source.
The images or other third party material in this Peer Review File are included in the article's Creative Commons license, unless indicated otherwise in a credit line to the material. If material is not included in the article's Creative Commons license and your intended use is not permitted by statutory regulation or exceeds the permitted use, you will need to obtain permission directly from the copyright holder.
To view a copy of this license, visit https://creativecommons.org/licenses/by/4.0/

We would like to thank all reviewers for their constructive feedback and provide a point-by-point response to the individual comments below.

Original Reviewers' comments are in black.

Our response is in blue, changes in the main manuscript and SI are highlighted in yellow.

REVIEWER COMMENTS

Reviewer #1 (Remarks to the Author):

In this paper Broich and co-workers suggest that IAV induces nanoscale cellular reprogramming when engaging/priming with cellular receptors, having an impact on AP-2 and actin dynamics. Both issues seem to be reasonable provided that IAV needs to get inside the cell via clathrin mediated endocytosis to reach late endosomes and acidic pH.

The authors implement a method to immobilize IAV viruses employing a glass substrate modified with silane PEG NHS and silane PEG (Figure 1) assuming that this way, one could recover the interactions between IAV receptros and AF (or other factors) and characterize them since, they are transient. The authors immobilize IAV viruses labelled with DiD. The same criticism made on the accompanying paper regarding the true nature of IAV and DiD not being the best marker for viruses (as it stains every vesicle, exosome or junk tht contains lipids) can be made here. I assume there is a subset of IAV particles that are also labelled with DiD but one cannot be sure unless staining both, the receptors and a viral component (matrix M1?). The different sizes for these particles could account on the fact that one can image aggregates, exosomes, viral IAV particles and junk that contains IAV receptors.

Response: The reviewer raises an important point. We routinely perform quality measures to validate the quality of our virus preparation and have now included this data into the manuscript. Since it has not been possible so far to generate an infectious IAV clone encoding a fluorescently-labeled M1 (see also response in accompanying paper Osman *et al*.), we and others have used membrane stainings to label intact IAV particles for live-cell imaging. To ensure that the DiD-labelled particles represent intact IAV particles, we immobilized our DID-labelled virus stocks on microscopy glass slides and performed immunostaining against the viral spike proteins (anti-H1) and the nucleoprotein NP. As now shown in the new Figure S1, we see that the majority of DiD-labelled particles indeed contain HA, which is important for our further experiments. For NP, we see a lower colocalization indicating free NP (e.g., vRNPs) in our preparation. We also performed negative-stain electron microscopy to further validate the presence of intact and spike-decorated particles in our preparation. We did not find indication for other vesicles or exosomes and thus assume they are not present in high numbers. Our TEM analysis also showed some particle pleiomorphy, which is expected for IAV (Harris, PNAS, 2006). Considering spherical viruses with a diameter between 100-200 nm (viruses are filtered using a 0.2 µm sterile filter), the viral surface area would scale between 0.12 μm^2 and 0.5 μm^2 (~5-fold). We thus think that the particle pleiomorphy at least partly accounts for the different sizes (i.e. DID intensities) observed.

In Figure 2, one can measure with super-resolution how EGFR accumulates around these particles, when tracking those employing MSD a bimodal distribution (mobile and immobile is seen). Not sure how these, or other receptors will adapt to particles decorated with different IAV receptors. In here, it is possible that the degree of incorporation plays a role. One needs to perform these experiments with IAV particles that are productive and one can recognize before hand. Technically, these experiments are of high complexity and seem to be performed really well and with care.

Response: As mentioned above, we performed and now include our viral quality analysis in the manuscript as new Figure S1. Concerning the point of other IAV strains, we have and still perform similar measurements with other human-pathogenic IAV strains. Measurements for H3N2/X-31 interacting with EGFR are shown as a Rebuttal Figure 1 for your reference. This data is part of another study and will thus not be included in the manuscript. Since EGFR was shown to be a glycoprotein involved in infection of H3N2/X-31 (Zhu, J Virol, 2014), we see similar behavior as observed for H1N1/PR8. We also want to point out that we investigated H1N1/PR8 interacting with MHCII in the accompanying paper by Osman *et al.* and did not see a pronounced slow-down of receptors as observed for H18N11.

[redacted]

The TIRF data shown in Figure 3 is also of high calibre. However, what these data show is that most likely, any particle decorated with IAV receptors will induce AP2 recruitment and will initiate CME; which in turn will have an impact on actin rearrangement (Figure 4). Also, even if the authors can show this for real IAV particles able to fuse in late endosomes, these facts were previously described with SVT.

Response: We thank the reviewer for this encouraging comment. Both the induction of endocytosis and rearrangement of actin are shown here as case studies of our newly developed assay. Clathrin recruitment was shown before (refs 7 and 22), but the local actin dynamics were, to the best of our knowledge, not investigated so far. The recruitment of AP-2 to the IAV binding site was not known before and is now part of our investigation with other IAV strains, as mentioned in the previous response.

Overall, the experiments in this paper are very well performed and the microscope approaches, together with image analysis and stats are very nice and rigorous. However, I think one needs to be certain that one is imaging real IAV particles. Also, it would be of interest to follow these particles with WF and check how they are entering the host, and if some of them are fusogenic at all. By combining DiD labelling with internal markers one should be able to tell which of these particles is undergoing hemifusion and full fusion as has been shown by others in the past.

Response: We thank the reviewer for the constructive feedback. We now present additional evidence showing that our DID-labelled particles are indeed intact viruses and, most importantly for our study, are decorated with viral spike proteins. With the current setup, the particles are covalently bound to the glass surface and thus we cannot follow their endocytosis/endosomal fusion. This immobilization was selected on purpose to synchronize all particles to the step of viral-cell attachment and thereby avoid ambiguity about the stage of infection. We are currently working on a new linker structure that can be cleaved during the acquisition and will allow us to follow particles during entry.

Reviewer #2 (Remarks to the Author):

The papers Christian Sieben describe the nanoscale clusters of influenza A virus receptors upon HA binding.

The data is compelling and of interest to a broad readership. The microscopy approaches are fantastic. Although the first manuscript is of a technical complexity, probably beyond a part of that broad readership. The capture of live cells using both virus and fibronectin comes across more as a trick, without real biological consequences. Would there be fluorescently labelled glycosylated membrane proteins that are not clustered upon engagement of the virus?

Response: We thank the reviewer for this assessment. We would like to clarify the use of fibronectin in both manuscripts since there might have been some confusion. Broich *et al.* describe a glass slide preparation in which viruses and cRGD are immobilized during the same step via reactive NHS. cRGD, a small circular peptide derived from fibronectin, was important to allow efficient attachment of cells to our otherwise pegylated glass surface (Broich *et al.*: Fig. 1D). In the paper by Osman *et al.*, we also started to use commercial slides functionalized with a 3D epoxy surface (PolyAn, Berlin). Epoxy residues can form covalent bonds with biomolecules and we found that here cRGD was not required for efficient cell attachment. Presumably, this is due to the underlying polymer matrix used to generate the 3D epoxy surface. Importantly, after virus immobilization, all remaining reactive epoxy groups are quenched before cells are added. Hence, cellular proteins are not immobilized by this approach. We have now added additional information in the methods section to clarify this point (Broich *et al.*, lines 459-462). The reviewer is correct that we did not observe any differences between the two virus immobilization approaches.
We expect that fluorescently labelled glycosylated membrane proteins that are e.g. differently localized at the cell membrane than EGFR are not clustered upon engagement of the virus. This is currently analyzed in a larger ongoing study and beyond the scope of this study.

This methodology was then applied to H18N11 in an accompanying paper. This virus uses a protein as a receptor and thus rules of engagement could be different, but in the end was not. In this paper the interaction of H18 and different MHC construct where first studied in detail before state-of-the-art microscopy approaches were used to dissect receptor clustering. Due to the availability of a null-mutant receptor protein clustering was convincing.

Eventually clustering of receptors appears to be quite universal, and this could be discussed more. It also decreases the novelty somewhat as it is quite expected and the technological advancements is then the novelty here.

Response: We agree with this conclusion and have extended the discussion (Broich *et al.*, lines 356-357).

Single IAVs induces Nanoscale cellular reprogramming at the virus-cell interface
Major
-The introduction steers towards a protein receptor, yet these are proteins that migrate from and to the plasma membrane, sialylated glycans on those proteins are the receptors. This change in premise should be be accounted for.

Response: We have modified the manuscript accordingly, now using the term *receptor* for the sialylated glycans.

-Fig 1D, sure viruses are located under the cell, but also next to it, there is no data for actual attachment as this could just

be due the the fibronectin peptide (which by it self is a smart move). The concluding text of this paragraph should be toned down.

Response: We agree that this could be a possibility and have thus modified the 2nd paragraph on page 5 (lines 155-159).

-The same holds true for the conclusion of the next "chapter", it is not known if this cluster is "functional". Again, probably glycans on EGFR are bound so it should say capable of clustering EGFR or something along that line.

Response: We agree and have modified the paragraph 3 on page 7 (lines 229-231).

-Only 15% of the viruses colocalize with actin? Is this a lot? What does this mean? It is gleaned over it, I also don't get the arc, blobs and holes classification.

Response: We apologize for not providing more details about our conclusions. From the STORM data of phalloidin-stained fixed cells, we classified the organization of filamentous actin at the virus-binding site into three groups 1) local enrichment (i.e., blob), 2) absence of signal (i.e., hole) or 3) ring- or arc-like structures. The live-cell imaging then revealed similar structures resembling these three groups, suggesting that they might be part of the same actin filament turnover process. Since the actin re-organization was very dynamic, as shown in Figure 4, the local enrichment was short-lived and only visible in 10-20% of the frames in the live cell movie. This observation is thus consistent with our observation in fixed cells considering a larger number of viruses and assuming similar actin turnover.

We thank the reviewer for this comment and have now extended the respective discussion in paragraph 1 on page 10 (lines 305-309).

Minor
-IAV viral envelope proteins are not spikes…

Response: We modified the wording accordingly.

-sialylated glycans are receptors and not attachment factors, please change throughout

Response: We modified the wording accordingly as mentioned above.

-the graphs Fig 1 B and C should be discussed and referenced in the text better, I had to go back and forth many times.

Response: We added references in paragraph 2 on page 4, paragraph 1 on page 5 (lines 130-131 and 136-137).

-Why would you compared fluorescent beads with fluorescent beads on glass surfaces?! Just that the coating is efficient?

Response: There might have been some confusion here, but we compared fluorescent beads with DID-labelled IAVs. This was to test the efficiency of the coating and the degree of particle aggregation in our samples and during immobilization. We looked at the intensity distribution and found that both samples show a single overlapping peak indicating that virus particles did not aggregate during the labelling and immobilization procedure.

-Because AP2 is reqruited it can also still be dispensable, so this is not so surprising.

Response: We agree, from the presence of AP2 at the viral attachment site, we cannot conclude that IAVs rely on this adaptor for CME as other adaptors were also shown to be involved. However, the recruitment of AP2 with dynamics resembling that of AP2-dependent CME indicates a positive involvement. But this needs to be tested for instance in AP2 knock-out cells. We have thus modified the wording accordingly (lines 346-348).

-First line of the discussion ◊ reqruitment of glycoproteins

Response: We modified the wording accordingly.

The bat IAV H18N11 induces nanoscale MHCII clustering upon host cell attachment
Major
- "inverse infection" while there is no infection, just binding…

Response: We now changed the name of the method to "inverted attachment".

-Was the addition of the fibronectin peptide necessary here as well?! This should be described in an accompanying paper.

Response: We apologize for the confusion. As described in the response to comment 1, we have used two different virus immobilization protocols in the two studies.

Minor
-discussion, bat and avian IAV both cluster a receptor for endocytosis is conserved for other viruses as well… this could be further discussed

Response: We now discuss this and propose adaptation of the "inverse attachment" approach in order to study other virus-receptor interactions (lines 270-275).

-hardly any information is given in the methods for the creation of plasmids for the soluble expression of MHC proteins

Response: We now describe the generation of the MHC-II expression plasmids in more detail in the material and method section.

-same goes for the inverse binding assay how where these viruses coated the glas slides and how was this controlled?

Response: We added a more detailed description of the coating to the respective chapter in the "material and methods" section.

Reviewer #3 (Remarks to the Author):

Review Report:

The paper titled, " Single Influenza A virus induce nanoscale …. " by Broich et al studies virus-receptor interaction and reports that viral receptors are not immobilized by the virus. They report that this process further leads to the accumulation of local receptor. The authors claim to study these processes using TIRF-STORM super-resolution microscopy. To my understanding, this is the only novel feature of the present work. Here are my major comments:

1. The statement, " … preserving the viruses' native state for uncompromised cell interaction " is not substantiated.

Response: We agree and changed the wording, now stating that our approach **"**Our method extends the observation time for virus-plasma membrane association while minimizing viral modifications, facilitating live imaging of virus-cell interactions." (lines 45-47).

2. The statement, " … viral receptors are not immobilized by the virus but rather slowed down … " is not supported by imaging data.

Response: We agree, this was an overinterpretation and we have changed the wording, now stating that "viral receptors show reduced mobility at the virus binding site" (lines 48-49).

3. Note that, the dynamic response requires time-lapse super-resolution imaging and related single molecule analysis.

Response: We agree with the reviewer, but want to point out that we have performed extensive live-cell super-resolution imaging as presented in Figure 3 B-E. The figure shows live-cell single-particle tracking (spt)PALM data of viruses interacting with live cells and represents one novel application of our virus immobilization approach. We see this as a particular strength to visualize virus-receptor interaction at the single-molecule level, which was not shown before.
To better visualize the time-lapse dynamics of EGFR at the virus-binding site, we have created two additional time-binned PALM super-resolution movies, which are now presented as new Supplementary Movies 1 and 2, with quantification in Figure S6. The dynamic response of EGFR clusters can be observed, indicating the exchange of EGFR between the virus interface and the remaining plasma membrane.

4. Studies carried out in the literature related to super-resolution study of Influenza A virus is completely ignored. Please see the following papers that clearly indicate clustering study of HA in cellular system.
- Nikki M. Curthoys et al., Influenza Hemagglutinin Modulates Phosphatidylinositol 4,5-Bisphosphate Membrane Clustering, Biophys J . 116, 893-909 (2019).
- S T Hess et al., Dynamic clustered distribution of hemagglutinin resolved at 40 nm in living cell membranes discriminates between raft theories, PNAS 104, 17370-17375 (2007).
- Raut P, Obeng B, Waters H, Zimmerberg J, Gosse JA, Hess ST., Phosphatidylinositol 4,5-Bisphosphate Mediates the Co-Distribution of Influenza A Hemagglutinin and Matrix Protein M1 at the Plasma Membrane. Viruses. 14, 2509 (2022).

Response: We have extended the discussion towards other super-resolution studies of IAV and have added the mentioned references.

5. On page #108, it is state that, "… indicating a direct and quantifiable interaction between receptor and virus". This is not demonstrated well.

Response: This statement refers to the reduced mobility and local accumulation (see also response to point 7) of viral receptors at the virus-binding site. Since this reduced mobility depends on sialic acid as in the case of IAV/PR8 or a functional receptor as in the case of H18/MHCII (accompanying paper Osman *et al.*), we believe that the reduced mobility and local receptor accumulation are a result of a specific interaction between virus and the cellular receptors. The difference in receptor mobility, the cluster size and receptor accumulation (now shown in Figure S5, see also response to point 7) are quantifiable metrics that can be used to assess the interaction between receptor and virus. However, at this point we can not rule out the possibility that other cellular factors within the virus-cell interface lead or at least contribute to the reduced receptor mobility and local clustering. We are currently investigating in a systematic study using different IAV strains how HA-receptor affinity modulates the three cluster metrics described above. The results will be part of a future manuscript.

We have extended the respective discussion on page 4, now stating "… indicating a direct interaction …".

6. In the super-resolution study, a lot of information is missing such as, localization plot, Fourier Ring Correlation, drift-correction etc.

Response: We thank the reviewer for raising this point. This information was indeed missing. We now present localization and clustering plots in the new Figure S4 together with respective resolution estimates. For the latter, we use Fourier Ring Correlation (FRC) as suggested by the reviewer but also image decorrelation (Descloux, Nat Meth, 2019), an alternative parameter-free method for estimating the resolution in diverse imaging datasets. New Figure S4 shows an example of EGFR-mEos3.2 (A and B) representative for the live-cell PALM data (Figure 4B-E) and another example of phalloidin-Alexa647 representative for the STORM data obtained in fixed cells as shown in Figure 4B.
Sample drift was corrected routinely as part of our standard localization processing routine using redundant cross-correlation (RCC) developed by Wang, Optics Express, 2014. We have now added more details about the drift correction procedure into the manuscript (lines 503-508).

7. The characteristics of local reporter accumulation such as, size of accumulates, its density, number per cluster and fraction of accumulated reporters need to be determined.

Response: We have now performed an extensive clustering analysis of our live-cell PALM datasets. The experimental details of the clustering analysis are now added to the manuscript (lines 502-506) and the results are presented in the new Figure S5. As mentioned in the previous response, an exemplary localization map and clustering result of EGFR-mEos3.2 are shown in the new Figure S4A. New Figure S5 shows the 1) cluster size, 2) fraction of clustered localizations, 3) the cluster density per μm^2 and the number of localizations per cluster, a proxy for the local accumulation of receptors. Overall, we find similar clustering characteristics as reported previously for EGFR in A549 cells (Sieben, PLoS Path, 2020). When we looked at the localizations overlapping with immobilized viruses, we found clusters that were significantly larger (25 vs. 45 nm) and had approximately 4 times more localizations (88 vs. 388). As we also describe in the accompanying paper for IAV H18/MHCII, we interpret such a strong local receptor accumulation as an indication for specific binding between virus and receptor.

Based on the above and my reading of the manuscript suggests that the manuscript is not mature enough for publication. More work along with single molecule analysis need to be done for drawing the conclusions. In addition, the authors need to look at reported super-resolution study on Influenza A virus.

Response: We have performed extensive additional analysis of our data, which is now added to the manuscript as three new supplementary figures and two movies. We would like to thank the reviewer for providing constructive feedback, which let us perform the additional cluster analysis that now provides additional evidence and quantification of virus-receptor interaction. We hope our work is now more complete and suitable for publication.

We would like to thank the reviewer for their feedback and provide a point-by-point response to the individual comments below.

Original Reviewers' comments are in black.

Our response is in blue, changes in the main manuscript and SI are highlighted in yellow.

REVIEWER COMMENTS

In response 2, what needs to be done is to carry out additional experiments to support the statement, which is clearly not done. Just changing the wording is not good enough. Even the new statement, "viral receptor show reduced mobility at the virus binding site " needs to be substantiated with experimental imaging data.

Response: *In the original version of the manuscript, we noted that receptors are not immobilized but rather slowed down. We agreed with the reviewer that this statement was imprecise, as demonstrating immobility using single-molecule imaging is challenging. The primary read-out of our sptPALM experiments is the diffusion coefficient of viral receptors, where we see a reduction at the virus binding site. We believe that our updated statement, "viral receptors show reduced mobility at the virus binding site" accurately reflects our measurements. Please also refer to our response to point 3 and* ***Rebuttal Figure 1****, where we present new data demonstrating similar effects with a different IAV strain.*

Response 3 requires time-lapse super-resolution data, which is not provided. This is essential for understanding dynamic response (how single molecules comes together to form clusters and the build-up of cluster) during the observation time. Unfortunately this is not provided.

Response: *For the investigation of virus-receptor interaction, we primarily rely on live-cell PALM super-resolution microscopy, where the sparse photo-activation of fluorescent mEos3 proteins is used to localize and track them over a short period before they photobleach (sptPALM). To allow precise localization, one pre-requisite of sptPALM is a sparse activation density so that only one photoactivated protein should emit light within a diffraction-limited spot (diameter ~300 nm). The virus-binding site is such a diffraction-limited spot and hence the nature of the measurement is not compatible with the direct visualization of multiple proteins at once as they come together to form a cluster. To still be able to visualize the dynamic developments at the virus-binding site, we reconstructed time-binned movies of our sptPALM data (shown in Supplementary Movies 1 and 2 and Supplementary Figure 6). Here, we combine many individual molecules, which allows the observation of cluster build-up as shown and quantified in Supplementary Figure 6.*

Further visualization of receptor clustering requires other super-resolution microscopy methods but we believe this goes beyond the scope of the present study. We have added a section in the discussion about current limitations and future work (see lines 357-365 and 376-381).

In response 5, the systematic study using different IAV strains how HA-receptor affinity modulates the three cluster metrics should have been carried out. This is essential for the present study and the claims in this study.

Response: *We conducted additional experiments with the IAV strain H3N2/X-31, which, like the IAV strain used in our study (H1N1/PR8), binds to and activates EGFR (Zhu, J Virol, 2014). Below, we present the corresponding dataset as* ***Rebuttal Figure 1****. In summary, we observed reduced diffusion of EGFR in the vicinity of both virus strains, although the clustering characteristics differed. While these differences may be attributed to varying affinities, further investigation is needed to precisely characterize the HA-receptor affinity, which we believe goes beyond the scope of the present study. Since the data presented in* ***Rebuttal Figure 1*** *are part of an ongoing investigation, we want to share them with the reviewer but will not include them in the manuscript.*

In response 6, the clusters are hardly visible from non-clustered molecules (see, Fig. S4A). In general, the clusters come more prominently and are quite distinct (see, the clusters in Ref. Nikki M. Curthoys et al., Biophys J . 116, 893-909 (2019).). What is needed is probably large incubation time for large clusters. The study needs further investigation.

Response: *We have expanded Figure S4A now including a plot of the localization density, which is exactly what is quantified by the clustering algorithm. We agree that scatter plots of localizations are not ideal for visually identifying density differences (i.e., clusters). A scatter plot, where each localization is color-coded according to its localization density, is more appropriate. The clusters are clearly visible in Figure S4A. However, we want to note that the appearance of localization clusters depends on the nature of the molecule being investigated. EGFR forms relatively small, mostly circular clusters, as we have also demonstrated previously (Sieben, 2020).*

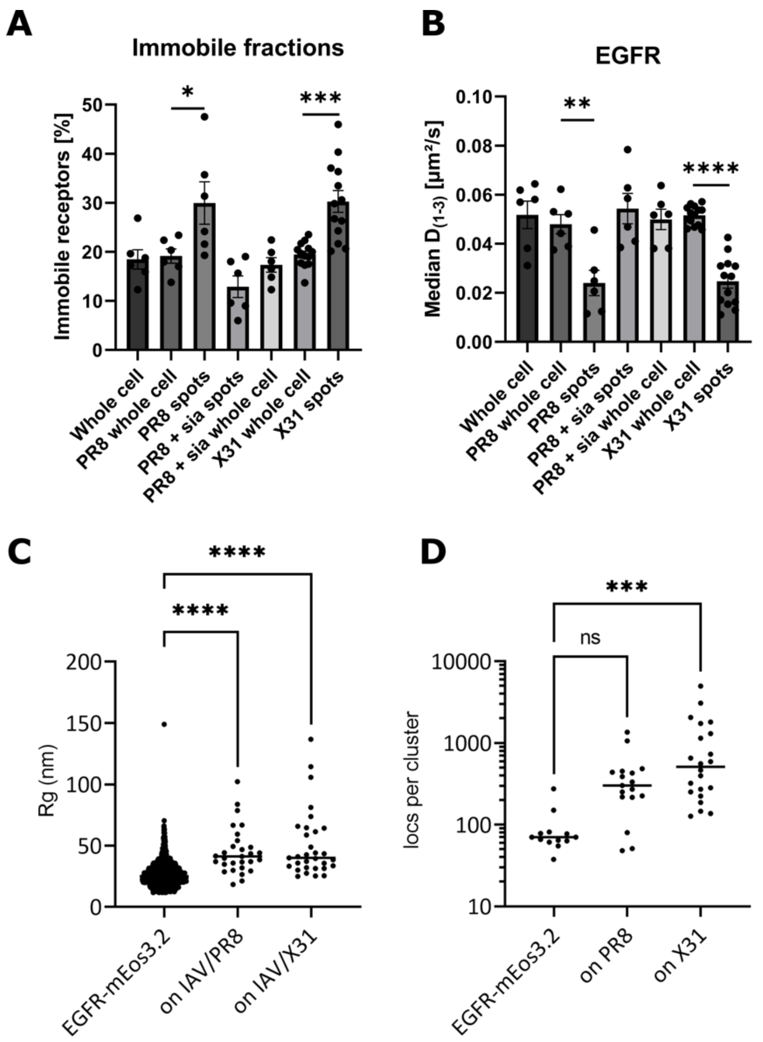

Rebuttal Figure 1: EGFR single-molecule characterization at the virus binding site. *EGFR immobile fraction (A) and diffusion coefficients (B) were measured in live cells in the presence of immobilized H1N1/PR8 or H3N2/X31. Clustering characteristics of single EGFR molecules at the binding site of immobilized H1N1/PR8 or H3N2/X31 viruses. The cluster size (C) and the number of localizations per cluster (D) were analyzed.*
